# Debris Flow Detection System Based on Tree-Topology Optical Fiber Network

**DOI:** 10.3390/s26144409

**Published:** 2026-07-11

**Authors:** Keiji Kuroda

**Affiliations:** Department of Physics, School of Science, Kitasato University, Sagamihara 252-0373, Kanagawa, Japan; kkuroda@kitasato-u.ac.jp

**Keywords:** debris flow sensor, optical fiber, time-division multiplexing, tree-topology

## Abstract

This report proposes a debris flow detection system based on an optical fiber network. Optical communication devices, including a distributed feedback laser, fiber amplifier, and amplified photodetector are used in the detection system. A tree topology fiber network formed by multiple optical couplers and delay fibers is employed to increase the number of sensor lines in the sensing port. Fiber patch cables or fiber mirrors are used as sensor heads instead of electric cables. The time-division multiplexing technique is used to simultaneously monitor all sensors in the time domain. It is demonstrated that this setup can be used to realize a passive and robust sensor network for debris flow detection by performing simulation experiments at the laboratory level. Results for a five-stage tree-topology are presented, and the extensibility of the proposed approach to a seven-stage network is outlined.

## 1. Introduction

In mountainous regions, natural disasters, such as landslides, rockfalls, debris flows, surface collapse, and snow avalanches can severely affect communities and infrastructure. To detect the occurrence of disasters as soon as possible and mitigate damages caused by them, studies have been focusing on developing early warning systems, and field tests have been conducted worldwide [1]. In observation stations, geophones, seismometers, ultrasonic sensors, electric wire sensors, and video cameras are used as sensing devices. Because each device has advantages and disadvantages, combined usage improves observation performance. Geophones, seismometers, and ultrasonic sensors are employed to detect ground vibrations caused by landslides and rockfalls, while temporal profiles and vibration spectra provide information on these phenomena [2,3]. Video cameras can directly observe visual images of debris flows to analyze their velocity and total material volume [4]. Wire sensors are stretched horizontally across rivers to provide warning signals when cut by debris flows [5,6,7]. By arranging multiple wires at regular intervals, the debris flow velocity can be calculated from the cutting times. In addition to data obtained by detection systems, precipitation and soil moisture observations are also used to predict debris flows [1].

Optical fiber sensors have attracted considerable interest because of their application to distributed and quasi-distributed large-scale sensing, including measurement of physical quantities, structural health monitoring, and civil engineering [8]. Various types of measurement techniques, light sources, sensor heads, sensitive detectors, and network topologies have been proposed and applied. To monitor the states of large-scale objects, multiplexing of the heads in sensor lines is essential. The number of interrogated sensor heads can be increased using techniques such as time-division multiplexing (TDM), wavelength-division multiplexing (WDM), and their combination. Although single line networks are simple and cost effective, they are vulnerable to issues such as the disconnection of fiber propagation. In topology networks, sensor heads are incorporated in independent parallel fiber lines connected using optical couplers or switches. If a fault occurs in one fiber line, the sensor heads in other fiber lines remain operational. Therefore, such topologies can be used to construct robust sensor systems. As independent sensor lines increase, the system’s robustness also increases. In contrast, an increase in the number of fiber couplers increases the optical loss. Therefore, a highly sensitive technique is required to construct a large-scale topology.

Typically, electric wire sensors are used for debris flow detection. Although the use of electric sensors is straightforward, it has certain shortcomings, such as limitations in transmission distance and multiplexing capacity. If electric wire sensors are replaced with an optical sensor network, devices and techniques developed in optical communication fields can be applied to realize a large-scale, long-distance, and robust debris flow detection system. Examples of optical sensor applications for geohazard monitoring can be found in the literature [9,10,11,12].

This report proposes a debris flow detection system based on optical fibers instead of electric wire sensors. A tree-topology network and the TDM technique allow simultaneous monitoring of 32 parallel sensing lines. Fiber patch cables are used as the sensor head. The system offers several advantages such as complete passivity, long-distance operation, and high temporal resolution. To verify the feasibility of the system, simultaneous detection of multiple fiber breakages in the network was performed by manually introducing cuts to selected sensing lines. Finally, it is demonstrated that the proposed method can be extended to 128 parallel sensing lines by replacing the patch cables with fiber mirrors.

## 2. Experiment and Results

### 2.1. Five-Stage Network

**Figure 1:**
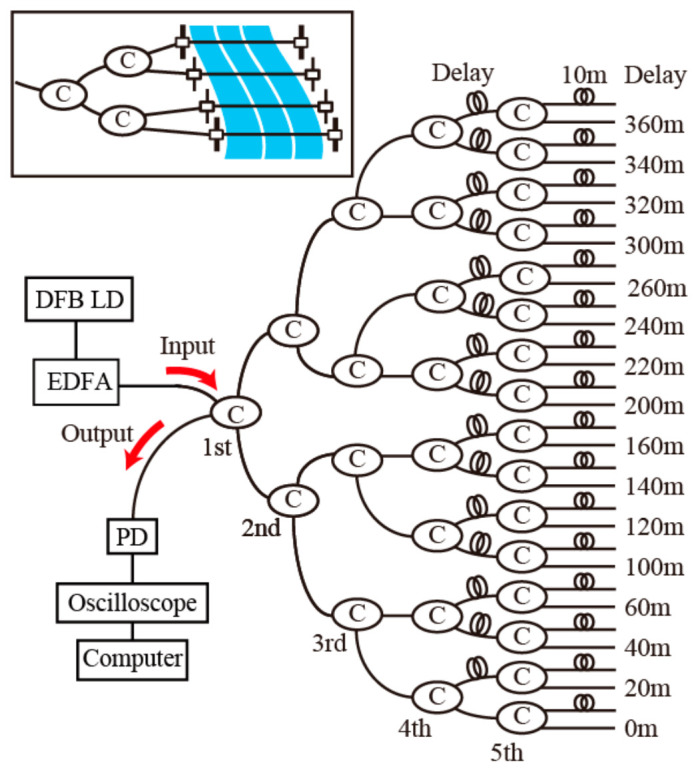
Schematic of experimental setup of five-stage tree topology. DFB LD: distributed feedback laser diode; EDFA: erbium-doped fiber amplifier; PD: photodetector; C: 50:50 coupler; inset: schematic of debris flow sensing system.

Figure 1 shows the experimental setup. The light source was a pulsed distributed feedback (DFB) laser (NLK1CMBLG, NTT Electronics, Yokohama, Japan). The laser’s oscillation wavelength was 1531 nm. The output power, pulse width, and pulse interval were approximately 5 mW, 70 ns, and 1 ms, respectively. The output pulses were introduced into the tree-topology network after being amplified by an erbium-doped fiber amplifier (EDFA) (AMP-FL8013-CBDB-13, FiberLabs, Saitama, Japan). Typically, EDFAs are used to amplify continuous light fields or short pulses with a high repetition rate. For such continuous or quasi-continuous operation, gain saturation is observed at the milliwatt input level owing to the slow recovery time of population inversion. When a narrow width pulse is introduced into the EDFA with a long interval time (>1 ms), a high gain exceeding 20 dB is maintained for an input power of 10 mW [13]. The reflections from the sensor heads were detected using an amplified photodetector (PDA10CS-CE, Thorlabs, Newton, NJ, USA) (PD) with a gain of 20 dB. Finally, the signals from the PD were monitored using an oscilloscope and stored on a computer. The results were analyzed to detect the disconnection of sensor fibers using the LABVIEW (LAVBIEW Ver. 12 National Instruments, Austin, TX, USA) program.

The sensor network consisted of a five-stage tree structure with 31 50:50 couplers. Without optical switches, 32 output ports can be used to connect the sensor heads [14,15]. The network was organized into four groups. In each group, eight sensor heads were connected in parallel using 10 m delay lines. The four groups were interconnected via 100, 200 and 300 m delay lines, respectively. A round-trip time of the 10 m delay is about 100 ns, which is longer than the pulse width of 70 ns. On the other hand, the pulse interval is set to 1 ms, which corresponds to a delay time of 100 km. Thus, reflections of the large number of sensing lines can be sequentially detected. This configuration enables temporal discrimination of reflections from sensor heads in different lines via TDM. The inset of Figure 1 shows a schematic of the debris flow sensing system used in a river. The 32 sensing fibers can be stretched over the river with an interval of 10 m. Successive downward fiber cuts are used to detect debris flow. Additionally, multiple sensing lines increase the survivability of the sensing station in the event of fiber breakage for reasons unrelated to debris flow. In contrast, the optical loss increases with the number of fiber couplers. In a multi-stage tree topology using 50:50 couplers, the reflection intensity decreases by a factor of (1/2)^2^ at each coupler. This is because both the input and reflection pulses traverse the couplers. The normalized intensity from the sensor heads connected to the output port of the n-th coupler is expressed as I = (1/2)^2n^. For the five-stage network, the normalized intensity is (1/2)^10^ = 0.00098. In Ref. [14], it was confirmed that the dependence of the cascaded coupler loss of the five-stage network followed the calculation. Additional losses induced by delay fibers are discussed later. The flat end of commercial physical contact patch cable is used as the sensor head. The reflections from the fiber ends are determined based on the Fresnel equation, as follows:

(1) $$R={\left(\frac{n_{g}-n_{a}}{n_{g}+n_{a}}\right)}^{2},$$

where *n*_g_ and *n*_a_ are the refractive indices of fiber glass and air, respectively. Assuming that the refractive indices of silica fiber and air are 1.4492 and 1.0003 [16], respectively, the reflectance at the air interface was calculated as 0.034. Therefore, to monitor whether the sensing fibers are connected or disconnected in the five-stage tree network, signals as weak as 10^−5^ of the input fields must be detected. As mentioned above, the total enhancement of signal detection using EDFA and PD is approximately 40 dB. This enhancement allows simultaneous monitoring of the connection/disconnection of sensing fibers with a sufficient signal-to-noise ratio (SNR).

**Figure 2:**
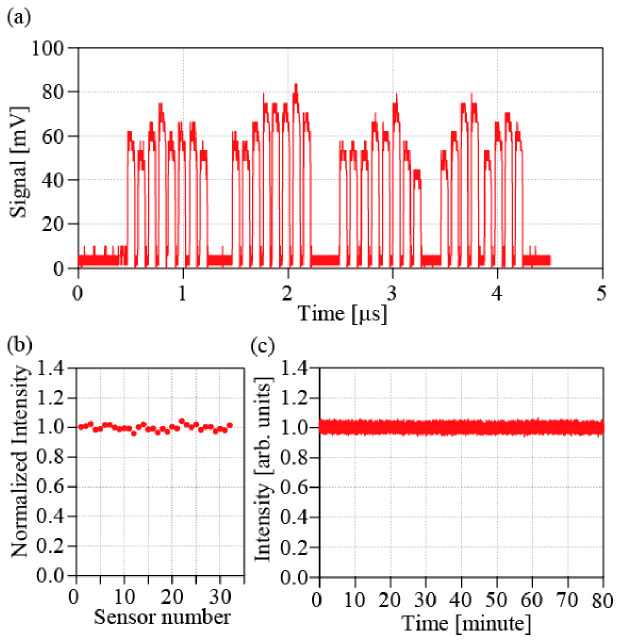
(**a**) Typical reflections; (**b**) normalized signal intensities of 32 sensor heads; (**c**) signal intensities of 32 sensor heads over 80 min.

Figure 2a shows typical reflections without averaging. By setting the pulse width to 70 ns, 32 pulses were clearly resolved in the time domain. The peak intensities of the pulses were distributed within a relatively wide range. Spliced fibers were used to adjust the delay times between the sensing lines. The optical loss at the splicing points may be different for each fiber, which may explain the intensity fluctuation. When a sensing fiber is disconnected, the pulse intensity decreases to the background level. Consequently, the distribution of signal intensities is not important for detecting disconnections. To monitor the states of the fibers, the intensities over a pulse width of 70 ns were summed, and the sum was normalized for clarity. These calculations were performed in the LABVIEW program. Figure 2b shows the normalized intensities for the 32 sensing lines. All intensities are distributed around 1.0. Figure 2c shows the normalized intensities of all sensors measured over 80 min. As can be seen, the intensities remain stable over the long term.

**Figure 3:**
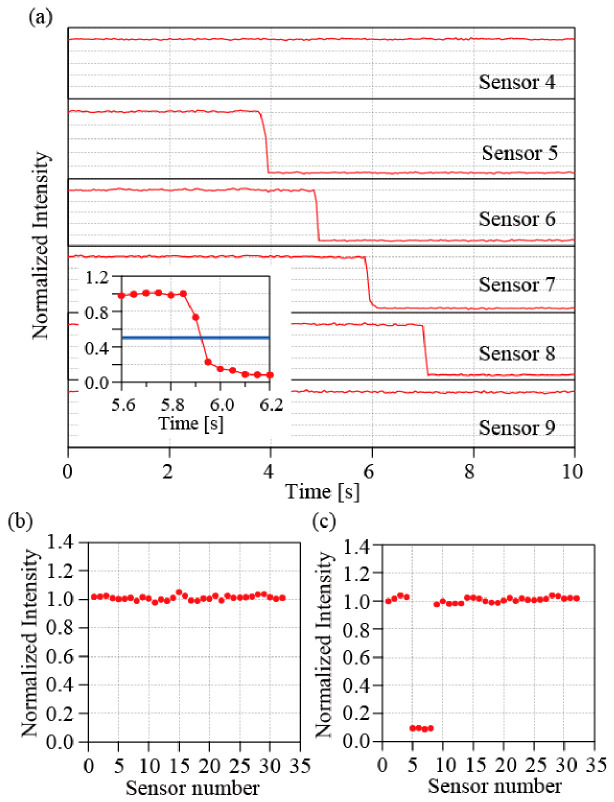
(**a**) Intensities of sensors 4–9 when disconnections are applied; inset: an enlarged view of the intensity variation in sensor 7. blue line indicates the threshold 0.5. (**b**) normalized signal intensities of 32 sensor heads at 2 s; (**c**) normalized signal intensities of 32 sensor heads at 8 s.

To assess debris flow sensing performance, disconnections were manually applied to sensors 5–8 with an interval of approximately 1 s. Because the interval of sensor lines is assumed to be 10 m, the expected speed of debris flows is approximately 10 m/s, which is the typical velocity observed in actual situations [7]. The intensities of sensors 4–9 are shown in Figure 3a. Abrupt decreases in intensity to the background level can be observed for sensors 5–8. The inset shows an enlarged view of the intensity variation in sensor 7. In this measurement, the sampling rate was set to 20 Hz. By selecting 0.5 (indicated by the blue line in the inset) as the threshold, the disconnection time was determined as 5.95 s for sensor 7. In the same manner, the disconnection times of sensors 5, 6, and 8 were determined as 3.95, 4.95, and 7.05 s, respectively. Figure 3b,c show the intensities of all sensors at 2 s and 8 s, respectively. The intensities of disconnected sensors can be clearly distinguished from those of connected sensors. Although a debris flow velocity is assumed to be 10 m/s here, the velocity may become faster depending on the terrains and the amount of water. In such situations, adjacent fibers might be cut almost simultaneously. However, fiber lines positioned at 20 m or 30 m intervals can be still used to measure the debris flow velocity without increasing the sampling rate, because the system has the large number of sensing lines. Based on the simulation results, it is expected that the system can be used to detect debris flow at multiple points and measure its velocity.

### 2.2. Seven-Stage Network

**Figure 4:**
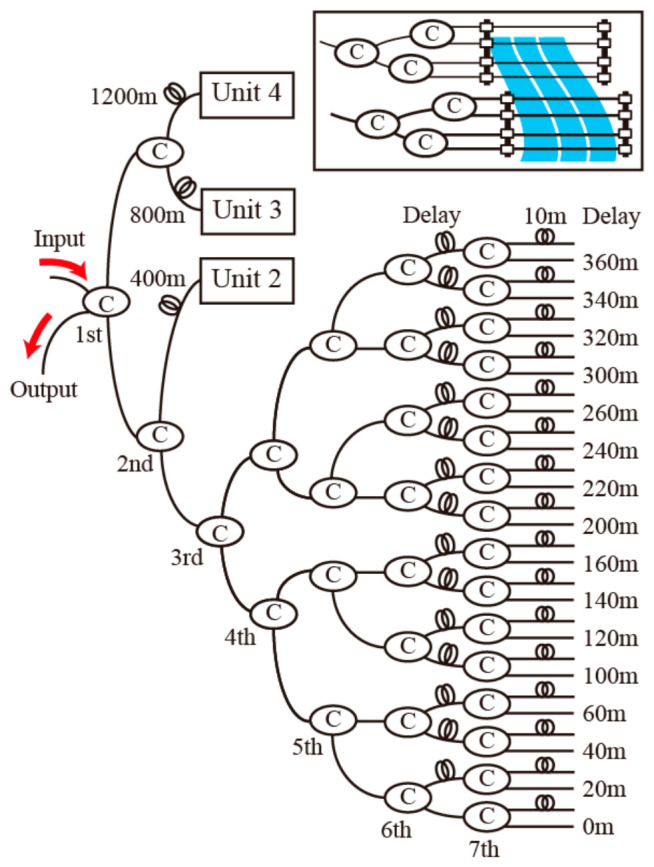
Schematic of experimental setup of seven-stage tree topology. C: 50:50 coupler; inset: schematic presentation of debris flow sensing system.

This subsection discusses the proposed system’s extensibility. Figure 4 shows the sensor network consisting of a seven-stage tree network, in which the five-stage tree network is treated as a unit. Because four units are connected in parallel by adding two-stage couplers, 128 lines are simultaneously interrogated. Here, only Unit 1 is connected as a proof of concept. In the seven-stage network, the propagation loss becomes (1/2)^14^. Consequently, the signal intensities decrease by a factor of 1/16 compared with those in the five-stage network. To compensate for the intensity decrease, fiber mirrors are used as the sensor head instead of the flat end of the patch cables. Because the reflectivity of the mirror exceeds 90%, the reflections increase more than 30 times. The delay length between each unit is 400 m. Assuming that the propagation loss of silica fibers is 0.2 dB/km, the additional loss for Unit 4 is 4% and does not induce severe decrease in the SNR. These estimations suggest that the seven-stage tree network can be realized without difficulty. The inset of Figure 4 shows a schematic of the debris flow sensing system based on the seven-stage network. Although it is possible to stretch the 128 lines with a 10 m interval, four sensing lines are treated as a single set to realize a more effective sensing network in actual situations. The four lines are placed at different heights at the same position with an interval of 10 m. These wires can detect the maximum depth based on the highest broken wire [17]. It is expected that additional information for analyzing debris flows can be obtained. Moreover, even if one of the wires breaks for reasons unrelated to debris flow, the system’s ability to detect debris flow is not compromised.

**Figure 5:**
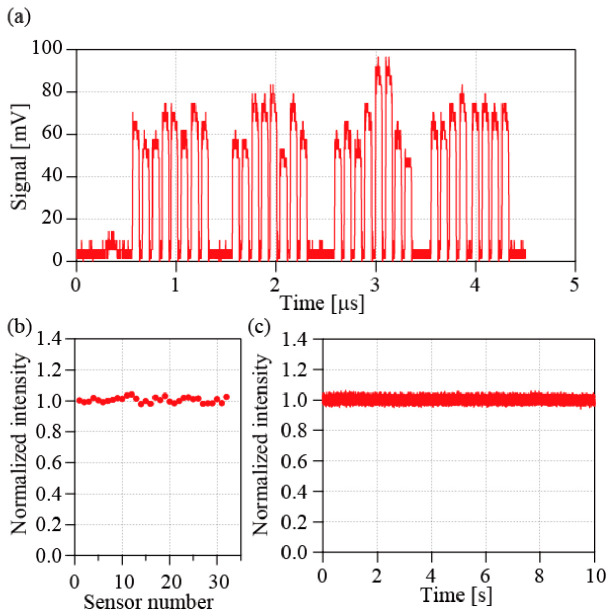
(**a**) Typical reflections in Unit 1; (**b**) normalized signal intensities of 32 sensor heads; (**c**) signal intensities of 32 sensor heads over 10 s.

Figure 5a shows typical reflections in the seven-stage network. Signals similar to those in the five-stage network are observed. The excitation of EDFA is adjusted to obtain approximately the same intensities as those in Figure 2a. Figure 5b shows the normalized intensities for 32 sensing lines. All intensities are distributed around 1.0. Figure 5c shows the normalized intensities of all sensors measured over 10 s. As can be seen, the intensities are stable. Although the fiber mirror is more expensive than fiber patch cable, the seven-stage network offers increased scalability and robustness compared with the five-stage network. Therefore, the seven-stage network is realizable using the same setup and procedure.

## 3. Discussions

In the five-stage network, 31 optical couplers and several delay fibers are required. Fiber patch cables or fiber mirrors are used as sensor heads. Although many components are required, they are standard commercial products and readily available. Notably, the network is fully passive and does not require an electrical power supply, and additional delay lines can be easily added. For example, the propagation loss of 1 km of fiber is approximately 9%, which can be readily compensated by increasing the excitation of the EDFA. Therefore, the monitoring station that includes all active devices, DFB LD, EDFA, and PD, can be installed at a safe position away from rivers. This is advantageous for realizing early warning systems in mountainous regions.

Electric wire sensors have been installed and used in monitoring stations in mountainous regions. Three wires stretched across the river at different heights were used to detect debris flow in Ref [5]. The scale of the debris flow is indicated by which wires are broken. In Ref. [6], eight wire sensors were installed to detect movements and measure velocities of debris flow. In Ref. [7], a single wire sensor was simply used to issue a signal that is broken when a debris flow passed through and broke the wire. When electric wires that are operated by independent systems are used, the clock of each system must be synchronized to measure the debris flow velocity. However, because the signal generator (laser) and detection device are shared among all sensing lines in the proposed setup, such synchronization is not required. In addition, a round-trip time of 1 km is about 10 μs, which is much shorter than the sampling time of 50 ms. Thus, a response delay at the monitoring station from the actual occurrence of a fiber cut is negligible. One drawback of wire sensors is the occurrence of false alarms triggered by accidental events [17,18,19]. In the proposed system, even if some wires are broken accidentally (passage of animals, falling trees), a series of wires with an interval of 20 or 30 m can still be used to detect debris flow and measure its velocity. When one reflection signal disappears, we cannot determine whether a disconnection accidentally occurs or a debris flow generates. However, a hazardous debris flow will sequentially cut successive fiber lines from upstream. Thus, we can distinguish the occurrence of debris flow from accidental phenomena and issue an alarm. To operate the system, we need to pay attention to some troubles like a disconnection in the tree topology. In my previous work, it has been confirmed that the observation of disappear/survival of reflection signals gives us information on fault positions [20]. These indicate that this system has robustness to troubles that may occur in actual situations. The tree-topology network makes it possible to link parallel sensing lines. Delay fibers with arbitrary lengths can be easily inserted in each line. These allow us not only to perform the large-area monitoring but also to apply TDM in the system. Thus, the measurements can be done by using a single-wavelength pulse source without an optical spectrum analyzer. In TDM technique, a short pulse with a long pulse interval is sufficient as the input field for the sensing. For the input of a narrow width pulse, the EDFA maintains a gain exceeding 20 dB [13]. This ensures the highly sensitive detection of the weak reflections by avoiding the saturation of the EDFA gain. The integration of well-established techniques, topology network, TDM technique and EDFA, allows us to perform simultaneous and large-scale sensing.

## 4. Conclusions

This report proposes a debris flow detection system based on optical fibers instead of electric wire sensors. A tree-topology network and the TDM technique are employed to simultaneously monitor 32 parallel sensing lines. Fiber patch cables are used as sensor heads. The system has the advantages of complete passivity, long-distance operation, and high temporal resolution. Simulation experiments were conducted by manually adding disconnections to some sensing lines. Based on the simulation results, it was confirmed that the system has the potential to detect debris flow at multiple points and measure its velocity. Additionally, the ability of the method to be extended to 128 parallel sensing lines was demonstrated by replacing the patch cable with fiber mirrors. In this study, simultaneous detection of multiple fiber breakages in the network was performed as preliminary experiments in laboratory. Thus, the environment was stable and the operation time was still short. The on-site tests under natural environment and over a longer term are needed to verify the feasibility of the method.

## Data Availability

Data can be provided upon request.

## Abbreviations

The following abbreviations are used in this manuscript:

TDM	Time-division multiplexing
WDM	Wavelength-division multiplexing
DFB-LD	Distributed feedback laser
EDFA	Erbium-doped fiber amplifier
PD	Photodetector
SNR	Signal-to-noise ratio
